# Fracture of the Neck of the Humerus

**Published:** 1851-09

**Authors:** 


*Fracture of the Neck of the Humerus.*—M. Lenoir presented to the Surgical Society of Paris a pathological specimen taken from the body of a woman aged 83, the patient having died three months after the accident. The nature or extent of the injury had remained doubtful. On examination of the body, a fracture of the anatomical neck of the humerus was found. The head of the bone had torn through the capsule, and was lodged in the subscapular fossa. A fracture of the surgical neck was nearly consolidated. The upper fragment was attached to the edge of the glenoid cavity by a portion of ligament. This cavity was nearly filled up by fibrous tissue.

MM. Maisonneuve and Larrey remarked upon the rarity of this kind of accident, and observed that its diagnosis is difficult.—*Dub. Med. Press.*

